# Effect of Pharmacist-Led Interventions on Medication Adherence among Vietnamese Patients with Asthma: A Randomized Controlled Trial

**DOI:** 10.3390/arm91030020

**Published:** 2023-06-13

**Authors:** Tan Thanh Nguyen, Mai Thi Xuan Truong, Dung Ngoc Lam, Tuyen Thi Thanh Le, Mai Tuyet Vi, Thanh My Tran, Thu Pham Minh Vo, Suol Thanh Pham, Bao Lam Thai Tran, Thang Nguyen, Lam Van Nguyen

**Affiliations:** 1 Faculty of Medicine, Can Tho University of Medicine and Pharmacy, Can Tho 94000, Vietnam; nttan@ctump.edu.vn; 2 Faculty of Pharmacy, Can Tho University of Medicine and Pharmacy, Can Tho 94000, Vietnam; truongthixuanmai17@gmail.com (M.T.X.T.); lamngocdung.d40@gmail.com (D.N.L.); maivivi127@gmail.com (M.T.V.); 1753030073@student.ctump.edu.vn (T.M.T.); vpmthu@ctump.edu.vn (T.P.M.V.); ptsuol@ctump.edu.vn (S.T.P.); nthang@ctump.edu.vn (T.N.); 3 Ba Ria Hospital, Ba Ria 78000, Vietnam; letuyen251287@gmail.com

**Keywords:** asthma, medication adherence, clinical pharmacists-led intervention, randomized controlled trial, Vietnam

## Abstract

**Highlights:**

**What are the main findings?**

- The proportion of nonadherence to medication in asthma patients was very high (22.3%), and patients’ behavior was the main reason (44.1%).
- Clinical pharmacist-led intervention in enhancing medication adherence in asthma patients, increasing treatment efficacy, relieving symptom severity, and reducing the medication burden from the disease.

**What is the implication of the main finding?**

- Intervention in medication adherence applied strictly following the education and counseling method would be beneficial for every healthcare practitioner to enhance the treatment outcome.
- Intervention in medication adherence should emphasize young patients and those with limited mobility.

**Abstract:**

Background: Medication adherence in asthmatic patients enhances the effectiveness of treatments, but some studies in low and middle-income countries still show some limitations. Our study aimed to determine if pharmacist-led interventions could increase medication adherence, improve treatment effectiveness, and relieve symptom severity in outpatients with asthma. Methods: We conducted a randomized, controlled trial on 247 asthmatic outpatients (aged ≥ 16) with a 1:1 ratio randomization at the hospitalization time and repeated after 1-month discharge. The primary outcome was to detect the difference in medication adherence between groups. Adherence was assessed by the general medication adherence scale (GMAS). Data collected by questionnaire was coded and entered into SPSS_20 for statistical analysis; Results: 247 patients (123 intervention, 124 control) were enrolled (61.1% male). After intervention, the adherence rate was higher among the intervention group than the control group (94.3% vs. 82.8%, *p* = 0.001). Patient behavior and knowledge were enhanced in the intervention group (*p* < 0.05). Asthma symptoms were relieved in the intervention group (*p* = 0.014). Pharmacist-led interventions on adherence rate were higher with OR: 3.550, 95% CI: 1.378–9.143, *p* = 0.009. Conclusions: pharmaceutical intervention could improve medication adherence, treatment efficacy, and the outcome should not be taken for granted; further research should be carried out in this regard.

## 1. Introduction

Asthma is a chronic respiratory disease characterized by coughing, wheezing, shortness of breath, and chest tightness. Asthma affects 339 million people around the world [1]. According to the National Health Interview Survey Data in the US (2019), up to 25 million people in the US have asthma, which is equivalent to 1 in 13 people [2]. The Global Asthma Report 2018 estimated that asthma is responsible for around 1000 deaths each day [1]. In Vietnam, 231,260 new cases of asthma annually, and the incidence-based economic burden of asthma was around VND 16,193 billion (USD 65,176) [3]. Asthma also puts many significant burdens on patients, such as years of living with disability, exacerbations, hospitalization, and medical costs, which in in low- and middle-income countries make this burden even more significant [1,4].

Drug treatments have demonstrated efficacy in reducing clinical morbidity and mortality. Although access to medication is necessary, it is not sufficient to successfully control the disease [5]. Therefore, adherence to medications for asthma is essential to enhance the effectiveness of management and improve health outcomes [6]. Treatment adherence contributed to patient cost savings and was consistent with the socio-economic situation in low- and middle-income countries [5]. However, several studies revealed that the adherence rate to asthma treatment is not high in practice (33.9%–41.9%) [7,8,9,10]. In Vietnam, few studies observed some problems in medication adherence, patient knowledge, and low level of improvement [11,12,13,14]. A cross-sectional study during the COVID-19 pandemic period found that the high requirement of pharmaceutical care in the community showed the vital role of pharmacists in medication guidance and education [15]. In addition, some systematic reviews have shown that the impact of an intervention on compliance generally diminishes over time [12,16]. Our study aimed to determine that clinical pharmacist intervention can improve adherence rates, increase treatment effectiveness, and reduce symptom severity in outpatients with asthma.

## 2. Materials and Methods

### 2.1. Study Design and Population

We conducted a randomized, controlled trial on 247 outpatients diagnosed with asthma, meeting the inclusion criteria at the Respiratory Clinic, Ba Ria Hospital, from 18 October 2021 to 10 January 2022.

Including criteria: outpatients with a diagnosis of asthma, patients aged 16 years or older, patients with old prescriptions at follow-up, and patients with complete administrative and paraclinical information.

Exclusion criteria: outpatients who did not agree to participate in the study, could not communicate in Vietnamese, and participated in a study of adherence within 1 year (up to the invitation timeline).

### 2.2. Sample Size

The sample size was estimated based on the study by Bruce G. Bender et al., 2010. The adherence prevalence in asthma patients after 10 weeks of intervention was 49.1% in the control group (p_1_ = 0.491) and 64.5% in the intervention group (p_2_ = 0.645) [17]. We calculated *n* = 215 with alpha = 0.05, beta = 0.1 (z_(α/2)_: 1.96 and z_β_: 1.282).

### 2.3. Data Collection, Randomization, Blinding, and Bias Control Method

Data were collected from medical records and patient interviews. All patients who participated in the study had signed informed consent forms. All patients were interviewed twice; the second time was 1 month apart from the first, collecting information about changes after the intervention. Counseling for the intervention group was conducted in the initial interview and 1 month after. The interview contents include (1) Patient information collection form; (2) Knowledge of diseases and drugs; (3) Compliance assessment using the general medication adherence scale (GMAS) (Table A1, Appendix A); (4) Assessment of symptoms of asthma control; (5) COVID-19 impact (non-adherence caused by COVID-19 due to social distancing). The consultation contents were suggested by 9 doctors and pharmacists with expertise in asthma.

We used a random sampling method by selecting all patients who met the criteria during the study period. The website (https://www.random.org/integer-sets/, accessed on 15 October 2021) decided on 247 random numbers, with the first 124 random numbers being the control group and the remaining 123 being the intervention group. By rearranging each group in ascending order we obtained 2 tables with random numbers. Patients at the enrolled time were numbered cumulatively during the study period. Based on the table we determined whether the patient belonged the intervention group or the control group. Our study designed an open trial with open information for pharmacists and patients.

Information bias may occur due to: (1) The patients not correctly remembering or understanding the question; (2) The interviewer not fully expressing the question’s meaning during the interview. Remedial measures include: (1) Clearly explaining the research purpose and calling for the support of the patients; (2) Questioning more clearly; (3) Guiding the patient to answer specific and detailed right at the beginning of the interview; (4) Directly explaining when requested; (5) Combining viewing medical records and prescriptions while interviewing patients or family members to understand the patient’s follow-up status and medications.

### 2.4. Research Outcomes

The primary outcome was to assess the pharmacist intervention effectiveness on medication adherence. Patients in the control group received routine care at the Hospital, including counseling and education from doctors, nurses, and pharmacists dispensing drugs. Patients in the intervention group received routine care, counseling, and education from the research team. The education and counseling content for patients of the research team consisted of 4 main parts: (1) Knowledge of diseases and drugs: providing information on the pathophysiology of asthma (name of the disease, cause, disease status, risk factors, symptom severity), exacerbation prevention, differentiating between controller and reliever (reliever), recognition and prevention of drug side effects, correcting inhalation technique errors, and correct storage of inhaler devices; (2) Advise based on the patient’s non-adherence reasons (according to the GMAS), emphasizing the role of adherence to inhaled corticosteroids (ICS) in reducing the risk of hospitalizations and death from asthma, especially for patients with mild asthma (steps 1 and 2), reminders to reduce forgotten medication intake, supplement knowledge, improve inhaler skills, increase adherence motivation. By joining the asthma club, patients could discuss the diseases, the benefits of drug cessation in symptom relief, disease progression, limiting exposure to individual risk factors, vaccination, diet, exercise, asthma prevention during exercise, and measures to prevent COVID-19 infection with doctors or pharmacists (directly or mobile); (3) Pharmacists also advise the patient’s doctors, discuss with the doctor about any encountered side effects, and choose the inhaler and spacer suitable for the patient; (4) Issuing documents for patients, the set of documents was built based on guidelines of The Global initiative against asthma 2020 (GINA) [18]. The intervention effectiveness was assessed between intervention groups (after the intervention) and the control group based on the following values: medication adherence prevalence, non-adherence reasons, ability to distinguish between control and reliever medicine, correct inhalation technique [19], and relief severity symptoms (Table A2, Appendix A) [18].

Secondary outcomes were (1) the medication adherence rate; (2) determining the associated risk factors. Adherence was determined based on Atta Abbas Naqvi’s general medication adherence scale (GMAS), which is a Likert scale consisting of 11 questions across 4 levels from 0 to 3 points. Medication adherence was divided into 5 levels, including high adherence (30–33 points), good adherence (27–29 points), partial adherence (17–26 points), low adherence (11–16 points), and poor adherence (0–10 points). Adherence rates were divided into 2 groups: non-adherence (GMAS scores < 30 points) and adherence (GMAS scores from 30 to 33 points) [19]. The GMAS lists 3 groups of non-adherence reasons, including (1) patient behavior (negative when total scores of questions 1–5 = 15), (2) comorbidities, medication burden (negative when total scores of questions 6–9 = 12), and (3) cost-related (negative when total scores of questions 10–11 = 6) [19]. Risk factors related to medication adherence include (1) pharmacist’s intervention (determined positive when there had been an intervention by a pharmacist in the education and consultation of patients); (2) age group (<60 years old and ≥60 years old); (3) education level (lower secondary school and high school or higher); (4) number of comorbidities (less than 3 diseases and 3 or more diseases [12]); COVID-19 pandemic impact.

### 2.5. Data Analysis

The data were statistically analyzed using SPSS 20.0 software. Descriptive statistics were used for qualitative variables. Ratios, mean, standard deviation (SD), median, and interquartile range (IQR) were used for quantitative variables. The association was assessed by univariate and multivariable logistic regression analysis, OR, 95% confidence interval (CI). We compared ratios using the chi-squared test. The results were statistically significant when the *p*-value ≤ 0.05.

### 2.6. Research Ethics

The protocol was approved by the Ethics Committee in Biomedical Research of Can Tho University of Medicine and Pharmacy (No. 02/PCT-HĐĐĐ dated 15 March 2021). All study participants, including patients or the family members of patients, voluntarily signed informed consent to participate with a clear understanding of data collection and use purposes.

## 3. Results

**Figure 1:**
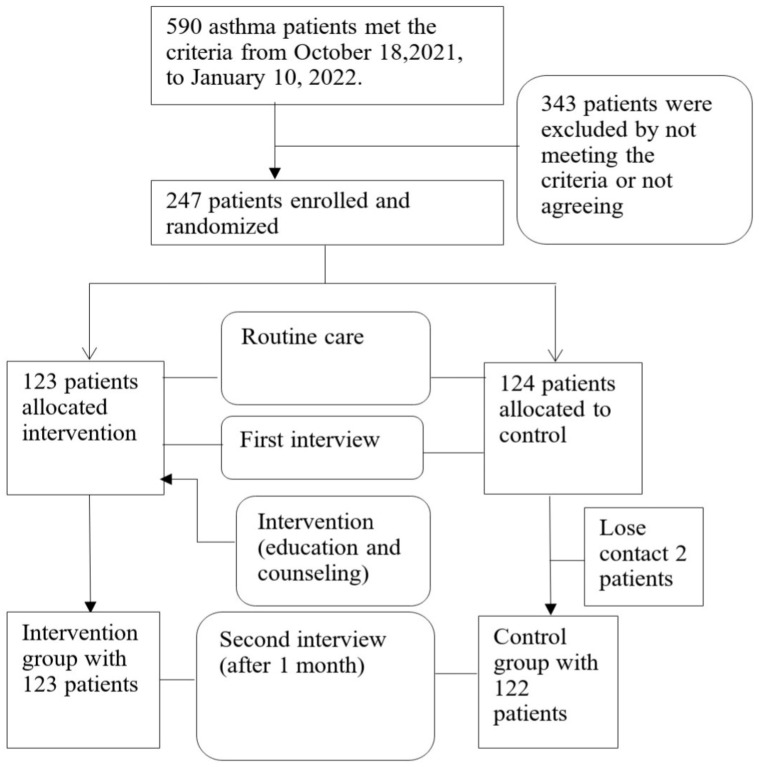
Study process flowchart.

From 18 October 2021 to 10 January 2022, 247 asthma patients met the criteria out of the 590 that were examined. A total of 343 patients were excluded due to not meeting the criteria or not agreeing to sign the informed consent. A total of 247 asthma patients were randomly divided into 2 groups: intervention (123 patients) and control (124 patients). After 1 month of interviews, the control group had 2 patients drop out (0.8%). Thus, the remaining 245 asthmatics were included in the analysis of drug adherence rates 1 month after counseling (Figure 1).

### 3.1. Baseline Characteristics of the Study Participants

Table 1 shows that there were 61.1% of males. The prevalence of ages over 60 and under 60 was approximately equal (49.4% and 50.6%). The median age in the study was 59 (51–67) years old, the lowest was 18 years old, and the highest was 94 years old. Patients with a high school degree or higher were 34.8%. There were 12.6% of patients impacted by COVID-19, 41.3% had risk factors, 13.0% were smokers, 53.4% step 3 of asthma treatment, and 35.2% step 4 of asthma treatment out of the total 247 enrolled patients. The difference in clinical characteristics between the intervention group and the control group was not statistically significant (*p* > 0.05).

### 3.2. Association of Pharmacist Intervention Effectiveness

#### 3.2.1. Intervention Efficacy on Adherence to Treatment

Table 2 shows that the adherence prevalence before intervention was 77.7%. The most important reason for non-adherence was patient behavior (44.1%). The difference between the intervention and control groups was not statistically significant (*p* > 0.05). After the intervention, the adherence rate in the intervention group was 94.3%, which is significantly higher than the control group (82.8%) (*p* = 0.005). Patient behavior was the most important reason for non-adherence (31.8%), and this prevalence was significantly lower in the intervention group (24.4% vs. 39.3%, *p* = 0.012). Comorbidities and medication burden were also significantly lower in the intervention group (*p* = 0.004).

#### 3.2.2. Intervention Efficacy on Asthma Symptoms and Patient Knowledge

Table 3 shows that the prevalence of well-controlled asthma symptom levels in the intervention group was significantly higher than in the control group (*p* = 0.014). There were no patients uncontrolled in the intervention group. Intervention improved patient knowledge due to distinguishing medication and correct inhalation technique, which is a significant difference between the two groups.

### 3.3. Risk Factors Associated with Adherence to Medication

Table 4 shows that pharmacist-led interventions on adherence rate were significantly higher with OR: 3.550, 95% CI: 1.378–9.143, *p* = 0.009. The COVID-19 impact reduced medication adherence with OR: 12.2, 95% CI: 3.02–50.00, *p* < 0.001.

## 4. Discussion

### 4.1. Principal Findings

Our study aimed to determine pharmacist-led interventions for increasing medication adherence, treatment effectiveness improvement, and symptom severity relief in outpatients with asthma. Before the intervention, the overall adherence rate for both groups was 77.7%, and there was no difference between the two groups. After the intervention, the adherence rate in the intervention group was significantly higher than the control group, showing that the intervention had increased the patient’s adherence rate (*p* < 0.001) (Table 2). The control group also received counseling from doctors and nurses, which could improve medication adherence in the control group. Multivariate regression analysis showed that pharmacist-led interventions on adherence rate were significantly higher with OR: 3.550, 95% CI: 1.378–9.143, *p* = 0.009 (Table 4). Non-adherence due to patient behavior was improved in the control group and significantly improved in the intervention group, showing that the intervention had enhanced the motivation of patient adherence (Table 2). The prevalence of well-controlled asthma symptoms in the intervention group was significantly higher than in the control group, indicating an improvement in symptom severity between the two groups. The intervention increased the patients’ knowledge based on distinguishing medication and correct inhalation techniques (Table 3).

### 4.2. Strengths and Weaknesses of the Study

Our study had a straightforward sample collection process with inclusion and exclusion criteria. All study participants volunteered and benefited from the study; asthmatic clubs were established to improve the adherence rate reduction over the period. The study design closely followed the checklists of CONSORT 2010. Research methods were clearly described and reproducible. The sample size was calculated clearly; therefore, data were specific to the Vietnamese population (*n* > 215; we had 247 patients, estimated 20% error). Our study showed a significant difference between the intervention group and the control group, clearly showing the role of clinical pharmacists in improving patient adherence, and thereby improving the treatment outcome, relieving symptoms, and reducing the disease burden. In addition, our study assessed several risk factors related to patient adherence. This could be a basis for policy reforms to improve adherence in patients.

However, there were also some limitations in our study. Our study was an open trial that was evaluated in one hospital. Therefore, it could bias the baseline characteristics, adherence rate, and treatment outcomes of the patients. A multicenter study with a larger sample size and blinding method is required for more accurate statistics. Our study assessed the adherence of patient within 1 month of follow-up, so the improvement was great and obvious. Adherence rates will likely decrease over time, so long-term follow-up studies are recommended for more accurate evaluation. Our study improved this limitation by organizing an asthmatic club. In the results section, we had not analyzed the difference in medication distinction and inhalation technique between each group’s baseline and follow-up period due to data loss. However, we found a significant difference during the study period that showed that pharmaceutic care enhanced the adherence and asthma symptoms of patients. In addition, we used a patient self-reported method, which may lead to bias due to unclearly remembering and underestimating medical use. This is a common method, but we still mentioned the improvement method in the bias control method.

### 4.3. Possible Explanations and Comparison with Other Studies

Before the intervention, the adherence rate (Table 2) was consistent with the studies of Ly T.T., 2019 and Entrenas Castillo M. et al., 2019, in which the adherence rate of asthmatic patients was 76.1%, and 70.67%, respectively (according to the TAI scale) [12,20]. The GMAS was developed in 2018, so the application was limited through previous studies. The adherence rate of our study was similar to a diabetes study that used the GMAS: 69% high adherence, 18.8% good adherence, 11.4% partial adherence, and 0.8% low adherence, and the GMAS median score was 31 (minimum of 11 scores, maximum of 33 scores) [21]. Our rate is higher than Ho N.N., 2019, Fernandez-Lazaro C.I., and Plaza V., 2019, with 34.4% (according to MMAS-8), 55.5%, 32.3%, respectively (according to the TAI scale) [9,13,22]. The difference in adherence rate may be due to the assessment methods difference, adherence assessment scale, and adherence level division (high compliance in the MMAS-8 scale is a maximum score of 8, for the TAI scale it is a maximum score of 50, while the GMAS is 30–33 points). In addition, it may be due to different patient characteristics, or the medication care at Ba Ria Hospital being relatively well performed, as there is an organized asthma club. The most important reason for non-adherence was the behavior of patients (Table 2), including discontinuing medication when feeling well, stopping taking medicine without telling the doctor, and forgetting to take medicine due to busyness (Table A3, Appendix A). Our study is consistent with the study of Ho N.N., 2019; the most important reasons are forgetting (42.0%) and stopping taking medicine when feeling well (32.0%) [13]. The study of Naqvi A. A. et al., 2019 on chronic disease patients showed a difference; behavioral reasons (33.9%) were lower than comorbidities and medication burden (49.1%) [23]. The differences were possible because of the population and socioeconomic status.

After the intervention, the adherence rate of asthma patients in the intervention group was significantly higher than the control group (Table 2), which is consistent with the study of Ly T.T. et al., 2019. The adherence rate of the study increased from 76.1% to 86% after 6 months of management [12]. The study of Wong, L.Y. et al., 2017 also showed similarities; the intervention group had a higher adherence rate compared with the control group (92.5% vs. 45.5%, *p* < 0.001) [24]. In the intervention group, there were no patients who made the wrong distinction between controller and reliever medication while the control group had 3.3% (*p* < 0.05) (Table 3), which is consistent with Adouni Lawani M. et al. (2018) and Fernandez-Lazaro C.I. et al., 2019 [22,25]. A study by Al-Awaisheh R.I. et al., 2023 used the inhaler technique scores to investigate the role of the pharmacist’s educational intervention in using the correct inhaler technique; the result showed similarly that the active group had a significant improvement in inhaler technique compared to the control group (*p* < 0.001) [26]. The intervention also reduced incorrect inhaler techniques in asthmatics (Table 3), which is consistent with the study of Wong, L. Y. et al., 2017 [24]. There was a significant improvement in asthmatic symptoms due to the increase in the proportion of well-controlled symptoms between the two groups. In the intervention group, no patients were without control (Table 3). The study of Ly T.T et al., 2019 also showed similar results; patients with good asthma control before treatment only accounted for 0.5%, but after 6 months this had increased to 4.7% (*p* < 0.05) [12]. The study of Al-Awaisheh R.I. (2023) used Asthma Control Test (ACT) and also found a similar result, the active group had a significant increase from 15.39 ± 6.136 to 21.21 ± 5.267 (*p* < 0.001) and much greater than the control group (*p* < 0.001) [26]. The study of Chinh Q. N. (2017) also showed similarities, which showed the prevalence of good control increased from 3.5% to 11.0% [14]. This rate is also consistent with the study of Wong L. Y. et al. (2017), in which the patients in the intervention group had better control than the control group (90% compared with 28.6%, *p* < 0.001), possibly because the patients in the intervention group had significantly better compliance and correct inhalation technique than the control group (*p* < 0.001) [24].

Our study showed that pharmacist-led interventions on adherence rate were significantly higher with OR: 3.550, 95% CI: 1.378–9.143, *p* = 0.009 (Table 4), which is consistent with the systematic review study by Jia X. et al., 2020. The study showed that pharmacist-led interventions positively affect the medication adherence and inhalation technique of asthmatics [27]. The adherence rate in the >60 age groups with OR: 2.345, 95% CI: 0.800–5.523, but there was no statistical significance (*p* > 0.05) (Table 4). A higher age is associated with adherence, which is consistent with the study of Dhruve H. et al. (2021) [28]. The COVID-19 pandemic reduced the adherence rate with OR: 12.2 (*p* = 0.001) (Table 4), which is consistent with the study of Fernandez-Lazaro C. I., 2019 [22]. There is a difference between the study of Kaye L. (2020) and Dhruve H. (2021); the adherence rate increased during COVID-19 from 53.7% (January 2020) to 61.5% (March 2020) [29], and increased from 34% (in 2019) to 42% (in 2020) [28]. This difference may be due to differences in medication availability across regions and countries. Factors such as gender, education level, occupation, smoking, comorbidities, and severe exacerbations in the past year were not statistically significant in multivariate analysis, which is consistent with Lemay J. et al., 2018, and Dhruve H. et al., 2021, and income and gender did not affect medication adherence [28,30].

## 5. Conclusions

Our study significantly shows the role of clinical pharmacist-led intervention in enhancing medication adherence in asthma patients, increasing treatment efficacy, relieving symptom severity, and reducing the medication burden from the disease. Risk factors related to medication adherence, such as pharmacist-led intervention, in the age group >60 had an increased adherence rate. In addition, the COVID-19 pandemic had a major impact and significantly reduced the adherence. These data could be the basis for policy reforms to improve the adherence rate. In addition, medication adherence intervention should emphasize young patients and those with limited mobility (such as preventing medical examination due to the COVID-19 pandemic). Therefore, the intervention will be beneficial for every healthcare practitioner to apply if strictly following the education and counseling content.

## Figures and Tables

**Table 1:** Baseline characteristics of the study participants.

Characteristic		Total (*n* = 247)		Intervention Group (*n* = 123)		Control Group (*n* = 124)		*p*-Value ^a^
		No.	(%)	No.	%	No.	%	
General characteristics								
Gender	Male	151	61.1	72	58.5	79	63.7	0.404
	Female	96	38.9	51	41.5	45	36.3	
Age	<60	125	50.6	61	49.6	64	51.6	0.751
	≥60	122	49.4	62	50.4	60	48.4	
	Median (IQR)	59 (51–67)		60 (49–67)		59 (51.25–67.75)		0.861 ^b^
	Lowest-Highest	18–94		18–94		20–92		
Education	None	8	3.2	3	2.4	5	4	0.751
	Primary school	49	19.8	22	17.9	27	21.8	
	Middle school	104	42.1	54	43.9	50	40.3	
	High school and above	86	34.8	44	35.8	42	33.9	
Occupation	None	115	46.6	58	47.2	57	46.0	0.516
	Workers, employees	46	18.6	25	20.3	21	16.9	
	Agriculture, Forestry, and fishery	31	12.6	11	8.9	20	16.1	
	Business	24	9.7	12	9.8	12	9.7	
	Others	31	12.6	17	13.8	14	11.3	
COVID-19 impacted	No	216	87.4	107	87	109	87.9	0.829
	Yes	31	12.6	16	13	15	12.1	
Clinical characteristics								
Risk factors	No	145	58.7	67	54.5	78	62.9	0.178
	Yes	102	41.3	56	45.5	46	37.1	
Smoking	No	140	56.7	68	55.3	72	58.1	0.064
	Stopped	75	30.4	44	35.8	31	25	
	Still	32	13.0	11	8.9	21	16.9	
Family history of asthma	No	190	76.9	97	78.9	93	75	0.471
	Yes	57	23.1	26	21.1	31	25	
Asthma treatment step	1	6	2.4	1	0.8	5	4	0.396
	2	7	2.8	4	3.3	3	2.4	
	3	132	53.4	63	51.2	69	55.6	
	4	87	35.2	48	39	39	31.5	
	5	15	6.1	7	5.7	8	6.5	
Severe exacerbations	None	214	86.6	102	82.9	112	90.3	0.088
	≥1	33	13.4	21	17.1	12	9.7	
Comorbidities	None	90	36.4	48	39.0	42	33.9	0.683
	1–2	99	40.1	48	39.0	51	41.1	
	3	58	23.5	27	22.0	31	25.0	

^a^: Using the Chi-square test, ^b^: Using the Mann-Whitney test.

**Table 2:** Patient adherence status before and after one month of intervention.

Characteristics		Total (*n* = 247)		Intervention Group (*n* = 123)		Control Group (*n* = 124)		*p*-Value ^a^
		No.	%	No.	%	No.	%	
Baseline								
Adherence rate (GMAS)	Non-adherence	55	22.3	24	19.5	31	25.0	0.300
	Adherence	192	77.7	99	80.5	93	75.0	
	Median (IQR)	32 (30–33)		33 (30–33)		32 (29.25–33)		0.487 ^b^
	Lowest-Highest	25–33		25–33		25–33		
Non-adherence reasons	Patient behavior	109	44.1	53	43.1	56	45.2	0.743
	Comorbidities and medication burden	54	21.9	24	19.5	30	24.2	0.373
	Cost-related	19	7.7	10	8.1	9	7.3	0.797
1 month								
Adherence rate (GMAS)	Non-adherence	28	11.4	7	5.7	21	17.2	0.005 *
	Adherence	217	88.6	116	94.3	101	82.8	
	Median (IQR)	33 (31–33)		33 (32–33)		33 (30.75–33)		<0.001 ^b,^*
	Lowest-Highest	23–33		25–33		23–33		
	*p*-value ^a^	<0.001 *		<0.001 *		<0.001 *		-
Non-adherence reasons	Patient behavior	78	31.8	30	24.4	48	39.3	0.012 *
	Comorbidities and medication burden	31	12.7	8	6.5	23	18.9	0.004 *
	Cost-related	12	4.9	6	4.9	6	4.9	0.988
	*p*-value ^a^	<0.001 *		<0.001 *		<0.001 *		-

^a^: Using the Chi-square test, ^b^: Using the Mann–Whitney test, *: statistically significant (*p* < 0.05).

**Table 3:** Asthma symptoms and patient knowledge before and after one month of intervention.

Characteristics		Total (*n* = 247)		Intervention Group (*n* = 123)		Control Group (*n* = 124)		*p*-Value ^a^
		No.	%	No.	%	No.	%	
Baseline								
Asthma symptom control level	Uncontrolled	16	6.5	10	8.1	6	4.8	0.409
	Partly controlled	104	42.1	54	43.9	50	40.3	
	Well-controlled	127	51.4	59	48	68	54.8	
Control and reliever medicine distinction	Correct	240	97.2	120	97.6	120	96.8	0.709
	Incorrect	7	2.8	3	2.4	4	3.2	
Inhalation technique	Correct	217	89.1	105	85.4	112	92.7	0.233
	Incorrect	27	10.9	18	14.6	9	7.3	
1 month								
Asthma symptom control level	Uncontrolled	2	0.8	0	0	2	1.6	0.014 *
	Partly controlled	69	28.2	26	21.1	43	35.2	
	Well-controlled	174	71.0	97	78.9	77	63.1	
	*p*-value ^a^	<0.001 *		<0.001 *		<0.001 *		-
Control and reliever medicine distinction	Correct	241	98.4	123	100	118	86.7	0.043 *
	Incorrect	4	1.6	0	0	4	3.3	
Inhalation technique	Correct	230	93.9	120	97.6	110	90.2	0.016 *
	Incorrect	15	6.1	3	2.4	12	9.8	

^a^: Using the Chi-square test, *: statistically significant (*p* < 0.05).

**Table 4:** Association between medication adherence and risk factors of the study participants.

Factors	OR	95% CI	*p*-Value
Pharmacist intervention			
No	1		0.009 *
Yes	3.550	1.378–9.143	
Age			
<60	1		0.075
≥60	2.345	0.919–5.986	
Education			
High school and above	1		0.453
Middle school and below	1.414	0.572–3.494	
Comorbidities			
<3	1		0.442
≥3	1.625	0.472–5.595	
COVID-19 impact			
No	1		<0.001 *
Yes	0.082	0.020–0.331	

*: statistically significant (*p* < 0.05).

## Data Availability

Not applicable.

## Appendix A

**Table A1:** General Medication Adherence Scale (GMAS).

	Question
1	Do you have difficulty remembering to take your medicine?
2	Did you forget to take your medicine due to a busy schedule such as travel, meeting, party, wedding, church/temple, etc.?
3	Did you stop taking the medicine when you feel well?
4	Did you stop taking the medicine when you experienced side effects such as stomach pain?
5	Did you stop taking your medicine without telling your doctor?
6	Did you stop taking medicine (for asthma) because you have to take more drugs for other diseases?
7	Did you find it inconvenient to remember to take your medication because of the complicated regimen?
8	In the past month, did you forget to take your medicine because of symptom severity and needed new medicine?
9	Did you arbitrarily change the drug regimen, such as dose, and times a day?
10	Did you stop taking your medication because the drugs were not worth the money?
11	Did you find it difficult to buy drugs because of their expenses?

Each question has 4 levels of choice: always (0 points), often (1 point), sometimes (2 points), and never (3 points).

**Table A2:** Asthma Symptom Control Level.

In the Past 4 Weeks, The Patient Had:		Well Control	Partly Control	Uncontrolled
More than 2 times symptoms a week in the daytime?	Yes □ No □	None	1–2	3–4
Night waking due to asthma?	Yes □ No □			
Need symptom relief medication more than twice a week?	Yes □ No □			
Activities limitation due to asthma?	Yes □ No □			

**Table A3:** Non-Adherence Reasons of Asthma Patients before Intervention.

GMAS	Total (*n* = 247)		Intervention (*n* = 123)		Control (*n* = 124)		*p*-Value ^a^
	No.	%	No.	%	No.	%	
Patient behavior							
1. Trouble remembering to take medication?	3	1.2	1	0.8	2	1.6	1.000 ^c^
2. Forgetting to take medicine due to busyness	64	25.9	34	27.6	30	24.2	0.536
3. Discontinuing medication when feeling well	74	30.0	32	26.8	42	33.1	0.178
4. Discontinuing medication when experiencing side effects	5	2.0	4	3.3	1	0.8	0.213 ^c^
5. Stopping taking medicine without telling the doctor	72	29.1	31	26.0	41	32.3	0.174
Comorbidities and medication burden							
6. Discontinuing medication due to using another medication for another disease	0	0.0	0	0.0	0	0.0	-
7. Inconvenient to take your medication because of the complicated regimen	7	2.8	3	2.4	4	3.2	1.000 ^c^
8. In the past month, discontinued because of symptom severity and needing new medicine	1	0.4	0	0	1	0.8	1.000 ^c^
9. Arbitrarily change the drug regimen, such as dose, and times of day	47	19.0	22	17.9	25	20.2	0.649
Cost-related							
10. Discontinuing because the medication is not worth	0	0.0	0	0.0	0	0.0	-
11. Trouble getting medication because of its cost	19	7.7	10	8.1	9	7.3	0.797

^a^: Pearson Chi-square, ^c^: Fisher’s Exact Test.

**Table A4:** Non-Adherence Reasons of Asthma Patients after The Intervention.

GMAS	Total (*n* = 247)		Intervention (*n* = 123)		Control (*n* = 124)		*p*-Value ^a^
	No.	%	No.	%	No.	%	
Patient behavior							
1. Trouble remembering to take medication?	3	1.2	1	0.8	2	1.6	0.622 ^c^
2. Forgetting to take medicine due to busyness	45	18.4	17	13.8	28	23.0	0.065
3. Discontinuing medication when feeling well	55	22.4	19	15.4	36	29.5	0.008 *
4. Discontinuing medication when experiencing side effects	2	0.8	1	0.8	1	0.8	1.000 ^c^
5. Stopping taking medicine without telling the doctor	54	22.0	19	15.4	35	28.7	0.012 *
Comorbidities and medication burden							
6. Discontinuing medication due to using another medication for another disease	0	0.0	0	0.0	0	0.0	-
7. Inconvenient to take your medication because of the complicated regimen	4	1.6	0	0.0	4	3.3	0.060 ^c^
8. In the past month, discontinued because of symptom severity and needing new medicine	1	0.4	0	0.0	1	0.8	0.498 ^c^
9. Arbitrarily change the drug regimen, such as dose, and times of day	26	10.6	8	6.5	18	14.8	0.036 *
Cost-related							
10. Discontinuing because the medication is not worth	0	0.0	0	0.0	0	0.0	-
11. Trouble getting medication because of its costs	12	4.9	6	4.9	6	4.9	0.988

^a^: Pearson Chi-square, ^c^: Fisher’s Exact Test, *: statistical significance (*p* < 0.05).
